# Class A G Protein-Coupled Receptor Antagonist Famotidine as a Therapeutic Alternative against SARS-CoV2: An In Silico Analysis

**DOI:** 10.3390/biom10060954

**Published:** 2020-06-24

**Authors:** Joseph T. Ortega, Maria Luisa Serrano, Beata Jastrzebska

**Affiliations:** 1 Department of Pharmacology, Cleveland Center for Membrane and Structural Biology, School of Medicine, Case Western Reserve University, Cleveland, OH 44106, USA; 2 Unidad de Química Medicinal, Facultad de Farmacia, Universidad Central de Venezuela, Caracas 1041-A, Venezuela; maria.serrano@ucv.ve

**Keywords:** antiviral therapy, G protein-coupled receptor, inhibitors, proteases, SARS-CoV2

## Abstract

The pandemic associated with Severe Acute Respiratory Syndrome Coronavirus type 2 (SARS-CoV2) and its disease named COVID-19 challenged the scientific community to discover effective therapeutic solutions in a short period. Repurposing existing drugs is one viable approach that emphasizes speed during these urgent times. Famotidine, a class A G protein-coupled receptor antagonist used for the treatment of gastroesophageal reflux was recently identified in an in silico screening. Additionally, a recent retrospective clinical report showed that the treatment with famotidine provided a good outcome in patients infected with SARS-CoV2. A clinical trial testing effectiveness of famotidine in combination with hydroxychloroquine is currently ongoing in the United States (US). In the 1990s, famotidine was described as an antiviral agent against human immunodeficiency virus (HIV). Interestingly, some HIV protease inhibitors are presently being used against SARS-CoV2. However, it is not clear if famotidine could be effective against SARS-CoV2. Thus, by using a computational analysis, we aimed to examine if the antiviral effect of famotidine could be related to the inhibition of proteases involved in the virus replication. Our results showed that famotidine could interact within the catalytic site of the three proteases associated with SARS-CoV2 replication. However, weak binding affinity of famotidine to these proteases suggests that a successful famotidine therapy could likely be achieved only in combination with other antiviral drugs. Finally, analysis of famotidine’s pharmacokinetic parameters indicated that its effect against SARS-CoV2 infection could be reached only upon intravenous administration. This work will contribute to the pharmacological knowledge of famotidine as an antiviral agent against SARS-CoV2.

## 1. Introduction

The Severe Acute Respiratory Syndrome Coronavirus type 2 (SARS-CoV2) pandemic known as COVID-19 has affected more than 6 million people worldwide as of June 2020 [1]. With an original epicenter in the Wuhan city in China, reported in December 2019, the virus spread all around the world in less than four months [2]. Since the outbreak began, several advances were made in the therapeutic management of SARS-CoV2 infection [3]. All these advances are associated with a better understanding of the viral pharmacological targets and the clinical outcomes of the applied treatments. We focused our interest on the proteases associated with the replication cycle of SARS-CoV2. The replication cycle of SARS-CoV2 involves two viral proteases, the main protease (3CLpro) and papain-like protease (PLpro), and one human host protease, transmembrane serine protease type-II (Tmprss2) [4,5,6,7]. The viral proteases play a fundamental role in SARS-CoV2 replication [8]. They are involved in the processing of the viral polyprotein into functional proteins critical for the viral life cycle. The cleavage motif characteristic for theses viral proteases is not present in the human host; thus, they could be used as specific targets in the viral pathogen for the development of pharmacological treatments [9]. These viral proteases of SARS-CoV2 belong to the family of cysteine proteases and exhibit high sequence homology to SARS-CoV proteases, with some structural modifications [10,11,12]. The main SARS-CoV2 protease has a larger surface within the substrate-binding site than the main SARS-CoV protease, potentially allowing for stronger interactions with the protease inhibitors [13,14]. These changes in the binding site could represent a therapeutic advantage for the development of new drugs or repurposing existing drugs. Drug repurposing offers multiple advantages over developing a new drug, including less risk of failure and lower cost; however, more importantly, it can accelerate the start of clinical trials, due to completed preclinical drug safety tests [15].

Previous reports indicated that human immunodeficiency virus (HIV) protease inhibitors display a strong antiviral effect, blocking the activity of the SARS-CoV main protease in vitro [14,16]. These results stimulated the search for compounds that could block the activity of the SARS-CoV2 main protease. Thus, based on data obtained by computational modeling and in vitro studies, indicating that HIV inhibitors such as lopinavir and darunavir could block the SARS-CoV2 main protease, several clinical trials were conducted to evaluate the efficacy of these drugs against SARS-CoV2 infection (NCT04321174, NCT04307693, NCT04315948, NCT04276688) [17]. However, the outcomes of these clinical trials were not as good as expected, likely due to the low bioavailability of these compounds, which prevented reaching the pharmacological target. Additionally, the ineffectiveness of these drugs could be related to the late stage of infection when these inhibitors were administrated to patients. In such a situation, when viral load was high, advanced pulmonary tissue damage could not be reversed. Moreover, therapy with these HIV protease inhibitors can produce well-known side effects [17]. Nevertheless, the main protease and two other proteases involved in SARS-CoV2 replication, the viral PLpro and the host protease Tmprss2, could be considered as targets to develop new anti-SARS-CoV2 therapy [9,18].

Recently, a clinical trial with famotidine, an antagonist of the histamine receptor type 2, belonging to the class A G-protein coupled receptor (GPCR) family, in combination with hydroxychloroquine was started (NCT04370262). Originally, hydroxychloroquine was developed as antimalarial drug; however, it was also later approved to treat autoimmune conditions. Hydroxychloroquine suppresses endosome-mediated viral entry to the host cells [19]. Famotidine was approved to treat gastroesophageal acid reflux; however, as discovered later, it also demonstrated some effectiveness in vitro in suppressing HIV. The mechanism of famotidine antiviral action was never clarified, but it could potentially act as a non-selective viral proteases inhibitor. Thus, the hydroxychloroquine–famotidine combination could produce a synergistic effect by blocking two different targets in the viral replication.

There are four Food and Drug Administration (FDA)-approved histamine type 2 receptor antagonists (H2 blockers): famotidine, cimetidine, nizatidine, and ranitidine to treat acid-peptic diseases, including duodenal and gastric ulcers, as well as gastroesophageal reflux disease [20]. While cimetidine and nizatidine are given orally, ranitidine and famotidine can be administered via both oral and intravenous routes, which is related to their chemical structures and pharmacokinetic parameters. Intravenous administration of famotidine could be advantageous to reach the tissue target where SARS-CoV2 is replicating actively. The mechanism of action of histamine H2 blockers is associated with their reversible binding to the active site of the histamine H2 receptor, which results in diminishing the H^+^/K^+^ pump activity, thus leading to lower acid production [20].

Currently, it is unclear whether famotidine, commonly used for the treatment of acid-peptic diseases, could block the activity of viral proteases. Thus, in this work, we performed an in silico molecular docking analysis to understand the potential of famotidine as an inhibitor of the key proteases involved in SARS-CoV2 replication. The obtained results were compared with other currently available viral protease inhibitors. In addition, we analyzed the pharmacokinetic profiles of these drugs to predict their successful use as anti-SARS-CoV2 therapeutics.

## 2. Materials and Methods

### 2.1. Protein Modeling

The homology structural model of human Tmprss2 (NP_005647.3) was built by using the tools of the SWISS-MODEL modeling server and the DeepView/Swiss-PdbViewer 4.01 software [21]. The best model for Tmprss2 was obtained using the crystal structure of serine protease hepsin with an inhibitor bound (Protein Data Bank (PDB) identifier (ID): 5CE1) as a template. Hydrogen atoms were added and partial charges were assigned for the energy refinement. The obtained model was subjected to molecular dynamic (MD) simulations using NAMD 2.12 [22], as described in Ortega et al. [23] using the CHARMM force field [24] and Gasteiger charges. The obtained structure represents the lowest energy frame of the MD simulations. The quality of the model was validated via ProSA-web [25] and PROCHECK programs [26].

### 2.2. Molecular Docking

The coordinates for the SARS-CoV2 main protease, papain-like protease, and HIV protease were obtained from the Protein Data Bank, PDB IDs 6LU7, 6WUC, and 3LZS, respectively. The PDB files were optimized by removing co-crystallized molecules and all crystallographic water molecules before being used for further computational analysis. Hydrogens were added and partial charges were assigned to all atoms. The obtained PDB files for each protein were further submitted to restrain the molecular mechanics refinement. All molecular dynamic simulations described in this study were performed with NAMD 2.12 [22] and VegaZZ 3.1.0.21 software [24,27] as described in Ortega et al. [23,28]. For each protein, the substrate-binding site located in the catalytic site was identified by using the Achilles Blind Docking server [29] with a respective ligand. The three-dimensional (3D) structure of each inhibitor was obtained from PubChem. Molecular docking was performed with VINA/VegaZZ 3.1.0.21 with 30 iterations for each compound. Results were prioritized according to the predicted binding free energy in kcal/mol. The results collected from the docking simulation were visualized via the Biovia Discovery Studio Visualizer 17.2.0 software.

### 2.3. Analysis of Pharmacokinetic Drug Properties

A comprehensive analysis of physicochemical descriptors, parameters related to administration, distribution, metabolism, and elimination (ADME), drug-like nature, and medicinal chemistry for famotidine and other protease inhibitors was carried out using SWISSADME tools [30]. These tools were accessed through the website at http://www.swissadme.ch. Moreover, a two-dimensional (2D) representation for the chemical structures of all drugs examined in this report are shown in Table 1.

## 3. Results

The viral and host proteases play a pivotal role in the SARS-CoV2 replication. The viral SARS-CoV2 genome codifies two proteases: the main protease (3CLpro) and papain-like protease (PLpro). Both enzymes were studied as possible targets for antiviral drugs [9,10]. As reported, HIV inhibitors such as lopinavir and darunavir could block the HIV main protease [14], while antivirals such as ribavirin, naphthalene derivative, and phenylthioacetic acid derivatives block the PLpro [31]. However, it is not clear whether these compounds could be used in combating the SARS-CoV2 infection. Thus, despite testing well-known antiviral drugs, the search for new anti-SARS-CoV2 therapeutics is urgently required. A drug-repurposing strategy provides an opportunity to discover new effects for already existing FDA-approved drugs, and it promises the shortest time to begin a clinical trial [15]. Retrospective clinical studies showed that famotidine, a histamine type 2 receptor antagonist, could improve the clinical outcome in patients diagnosed with COVID-19 [32]. In addition, recent computational analysis of therapeutic targets for SARS-CoV2 reported that famotidine could be a hit compound. [33]. Thus, we hypothesized that famotidine can inhibit the proteases involved in the viral replication. To gain a deeper understanding if famotidine indeed can interact with the catalytic site of these enzymes, we performed detailed in silico analysis.

### 3.1. Main Protease

**Figure 1:**
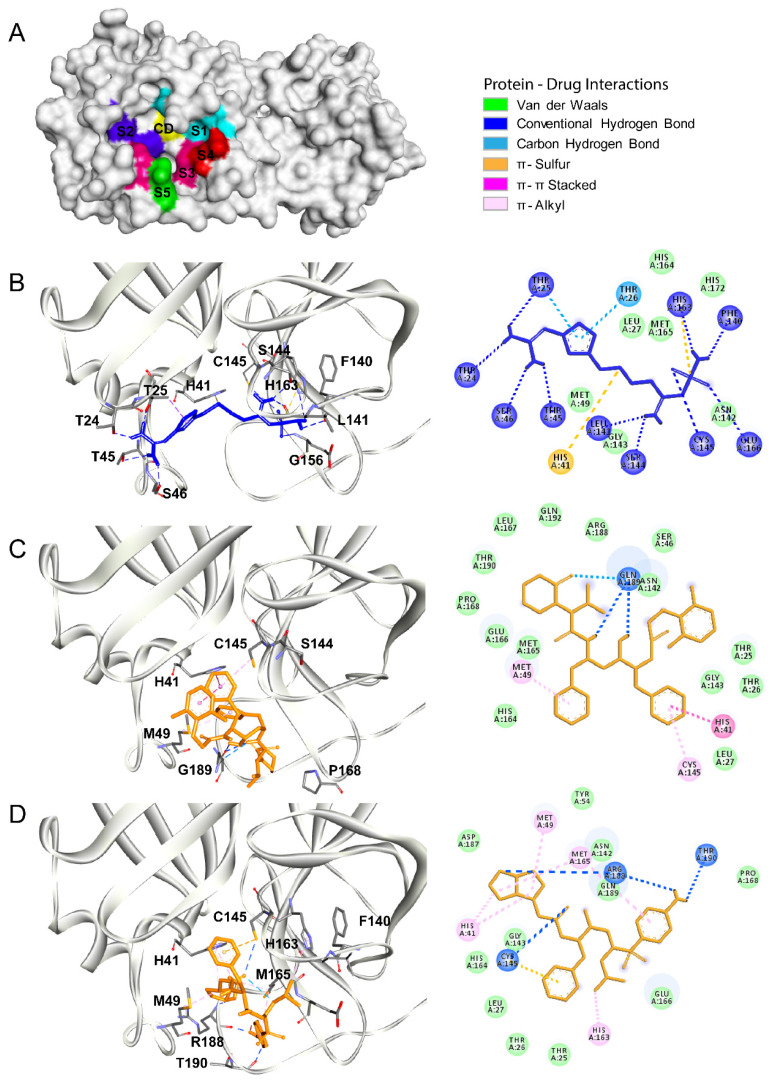
The Severe Acute Respiratory Syndrome Coronavirus type 2 (SARS-CoV2) main protease as a drug target. (**A**) The surface representation of the three-dimensional (3D) structure of the SARS-CoV2 main protease (Protein Data Bank (PDB) identifier (ID): 6LU7). The structural subdomains are indicated with different colors. The catalytic domain (CD) with the His41/Cys145 dyad is shown in yellow. Subdomains S1 (His163, Glu166, Cys145, Gly143, His172, and Phe140), S2 (Cys145, His41, and Thr25), S3 (Met165, Met49, and His41), S4 (Met165 and Glu166), and S5 (Gln189, Met165, and Glu166) are shown in cyan, dark blue, magenta, red, and green, respectively. (**B**–**D**) Molecular docking of famotidine, lopinavir, and darunavir into the catalytic site of the SARS-CoV2 main protease. The 3D structures of the protein–drug complexes are shown on the left. The protein is represented in ribbon, and drug ligands are shown as sticks. Famotidine is shown in dark blue, while lopinavir and darunavir are shown in orange. The main interacting residues are also indicated. The 2D diagrams of the main interaction networks between the drug ligand and the residues within the protease active site are shown on the right. The specific types of interactions are indicated in the legend. The final 3D structures and 2D diagrams of the protein–drug complexes were visualized with the Biovia Discovery Studio Visualizer 17.2.0 software.

The main protease, also named chymotrypsin-like protease (3CLpro), of SARS-CoV2 shares ~95% homology with 3CLpro of SARS-CoV. Structurally, these proteases are also very similar, showing only subtle differences (root median squared of 2.75, in the 306 Cα atoms) [34]. Moreover, both enzymes functionally belong to a cysteine protease group. The SARS-CoV2 main protease has a binding site with a catalytic dyad formed by His41 and Cys145, and several subsites designated as S1 (His163, Glu166, Cys145, Gly143, His172, and Phe140), S2 (Cys145, His41, and Thr25), S3 (Met165, Met49, and His41), S4 (Met165 and Glu166), and S5 (Gln189, Met165, and Glu166) (Figure 1A). The main protease is responsible for the proteolysis of pre-proteins associated with the viral replication machinery such as RNA-dependent RNA polymerase, a helicase, and an exoribonuclease, among others. Prior reports showed that anti-HIV drug lopinavir could block the activity of both SARS-CoV and SARS-CoV2 proteases [16,35,36]. Thus, in this work, we used a priori docking with lopinavir to define the substrate-binding site in the SARS-CoV2 main protease [14,37]. After determining the binding site coordinates, we analyzed the binding pattern and binding free energy of famotidine to the main SARS-CoV2 protease. The results obtained for famotidine were compared with two HIV protease inhibitors, lopinavir and darunavir (Table 2). Both lopinavir and darunavir showed lower binding free energy (−9.0 kcal/mol and −8.3 kcal/mol, respectively) as compared to famotidine (−6.4 kcal/mol), which is likely related to the chemical features of each inhibitor. Nevertheless, each compound (famotidine, lopinavir, and darunavir) could interact with the key residues, His41 and Cys145, related to the catalytic activity of the main protease (Figure 1B–D). Lopinavir and darunavir were able to produce hydrogen bonds, π–π, and π–sulfur interactions, with Hys41 and Cys145 residues of the catalytic center and with residues of the subsites S1, S3, and S5 (Met49, His54, His163, Met165, Pro168, Arg188, Gln189, and Thr190) (Figure 1C,D, respectively). Famotidine adopted a distinct binding mode as compared to lopinavir and darunavir. It also produced hydrogen bonds, π–π, and π–sulfur interactions with residues of the catalytic center (Hys41 and Cys145), but it interacted with different residues of the subsites S1, S2, and S4 (Thr24, Thr25, Hys41, Thr45, Ser46, Phe140, Leu141, Ser144, Cys145, Hys163, and Glu166) (Figure 1B). The detailed interaction patterns of lopinavir, darunavir, and famotidine with the main protease are shown in the 2D diagrams in Figure 1.

### 3.2. Papain Like Protease

**Figure 2:**
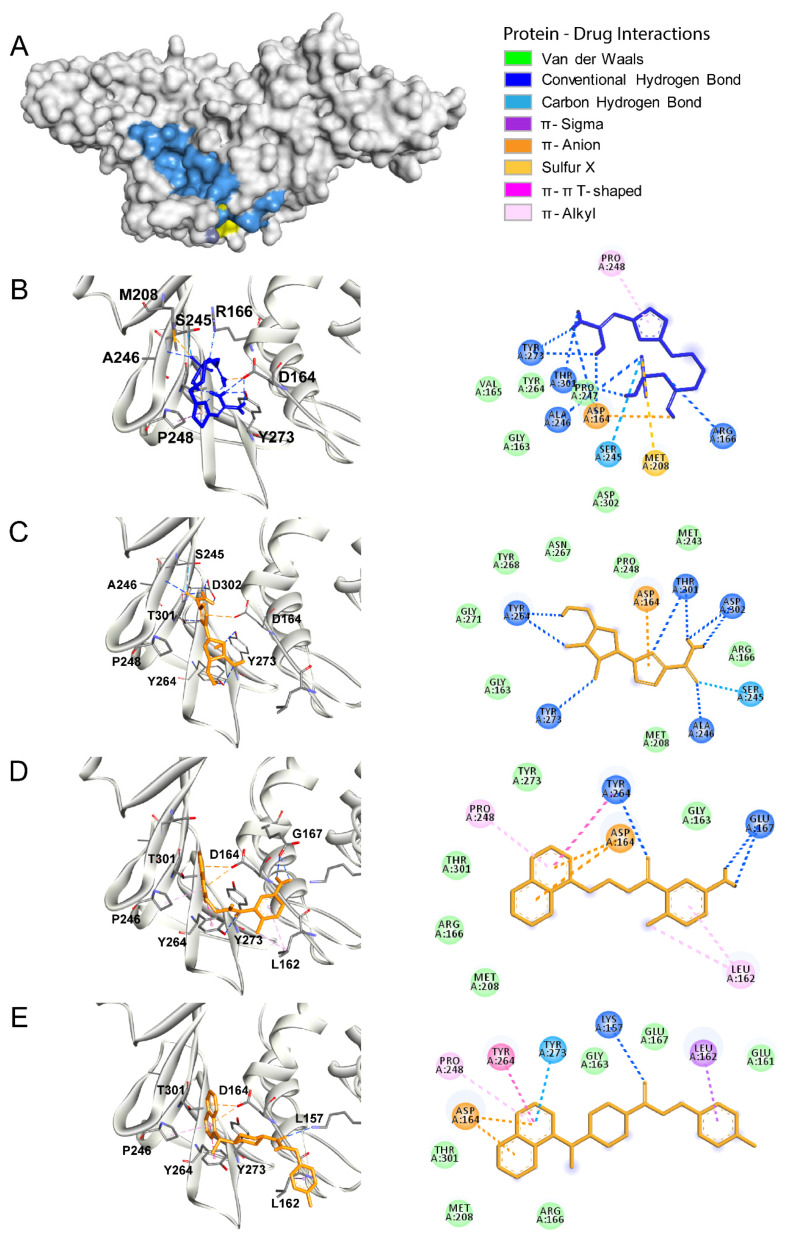
The SARS-CoV2 papain-like protease (PLpro) as a drug target. (**A**) The surface representation of the SARS-CoV2 PLpro crystal structure (PDB ID: 6WUC). The catalytic site is shown in blue and the catalytic triad is shown in yellow. (**B**–**E**) Molecular docking of famotidine, ribavirin, compound SARS-CoV(1), and compound SARS-CoV(2), respectively, into the catalytic site of the SARS-CoV2 PLpro. The 3D structures of the protein–drug complexes are shown on the left. The protein is represented in ribbon, and drug ligands are shown as sticks. Famotidine is shown in dark blue, while ribavirin, compound SARS-CoV(1), and compound SARS-CoV(2) are shown in orange. The main interacting residues are also indicated. The 2D diagrams of the main interaction networks between the drug ligand and the residues within the protease active site are shown on the right. The specific types of interactions are indicated in the legend. The final 3D structures and 2D diagrams of the protein–drug complexes were visualized with the Biovia Discovery Studio Visualizer 17.2.0 software.

The papain-like protease (PLpro) is also a cysteine protease. However, in contrast to the main protease, PLpro possesses an Asp/His/Cys catalytic triad within the substrate-binding site critical for its catalytic activity (Figure 2A; the active site is shown in blue and the catalytic triad is shown in yellow) [31]. These residues (Asp286, His275, and Cys111) are located within two main domains of PLpro. Cys111 is located within the central domain, which is mostly composed of α-helices, while His275 and Asp286 are located in the C-terminal region, which is composed of a β-sheet [31]. PLpro is responsible for the cleavages of the N-terminus of the polyprotein to release Nsp1, Nsp2, and Nsp3, non-structural proteins associated with the viral replication [38]. Furthermore, PLpro also has other functions such as de-ubiquitination and de-ISGylation (ISG: interferon-stimulated gene) related to antagonizing the host’s innate immunity. Compounds described as inhibitors of the SARS-COV PLpro could potentially inhibit the activity of the SARS-CoV2 PLpro [39]. Thus, we performed the molecular docking analysis to determine the binding free energies and the binding pattern of such compounds to the SARS-CoV2 PLpro, and we compared the results to those obtained for famotidine. Firstly, using the crystal structure of PLpro (PDB ID: 6WUC) and Castp 3.0 software, we performed topological analysis of the substrate-binding pocket of the SARS-CoV2 PLpro [28]. This pocket exhibits a solvent-accessible area of ~60 Å and it involves the following residues: Trp106, Asn109, Cys111, Tyr112, Leu162, Gly163, Arg166, Met208, Ser245, Ala246, Tyr264, Gly271, His272, Tyr273, Thr301, and Asp302. Next, we performed a priori docking using a common antiviral drug ribavirin as a ligand to confirm our analysis and to define the binding site coordinates. Then, we performed molecular docking of famotidine, ribavirin, and two other compounds described as the SARS-CoV PLpro inhibitors (Figure 2B–E, respectively; Table 3). Our results indicated that all the evaluated compounds could interact in the substrate-binding site via several electrostatic interactions and hydrogen bonds. Ribavirin mainly interacted with the following residues: Asp164, Ser245, Ala246, Tyr264, Tyr273, Thr301, and Asp302 via van der Waals interactions, hydrogen bonds, salt bridges, sulfur–x, and π–alkyl interactions. The binding free energy of ribavirin to the SARS-CoV2 PLpro was −6.1 kcal/mol, while the SARS-CoV inhibitors showed slightly lower binding free energy (−6.4 kcal/mol). The main interactions of these inhibitors with the SARS-CoV2 PLpro involved the following residues: Leu162, Asp164, Pro248 Tyr264, Tyr273, and Thr301. Among the analyzed compounds, famotidine exhibited the highest binding free energy (the lowest affinity) of −5.0 kcal/mol. The main interactions between famotidine and the SARS-CoV2 PLpro within the binding site involved the following residues: Asp164, Arg166, Ser245, Ala246, Pro247, Pro248, and Tyr273, and they occurred mostly via hydrogen bonds. All the interactions between the SARS-CoV2 PLpro and the inhibitors evaluated in this work are shown in the 2D diagrams in Figure 2.

### 3.3. Tmprss2

**Figure 3:**
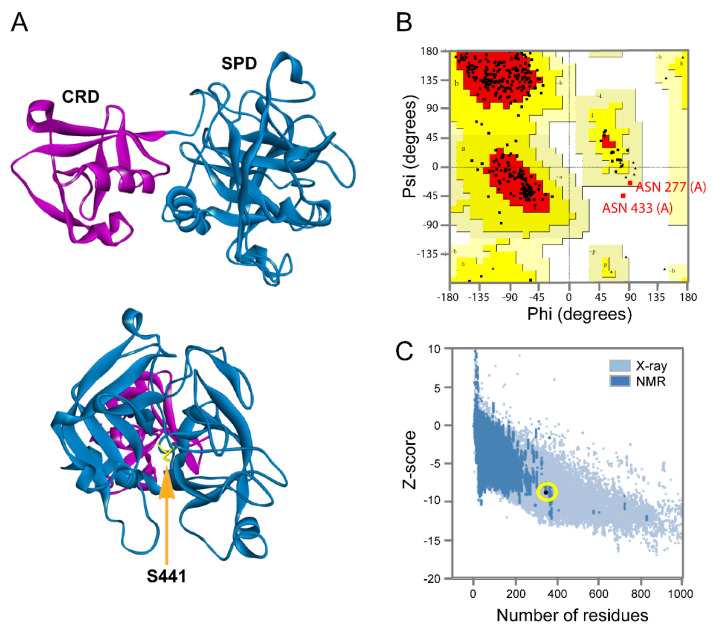
The homology model of human host transmembrane type 2 protease (Tmprss2). (**A**) The structural representation of the homology model obtained for human host protease Tmprss2 using the coordinates of hepsin protease (PDB ID: 5CE1) as a template. The protein has two main domains: the cysteine-rich domain (CRD), containing the residues 148–242, shown in magenta and the serine protease catalytic domain (SPD), containing the residues 255–489, shown in blue (upper panel). The front view of the catalytic domain. The key catalytic residue Ser441 is highlighted in yellow and indicated with an arrow. (**B**,**C**) The overall quality assessment of the Tmprss2 homology model using the Ramachandran and ProSA programs.

**Figure 4:**
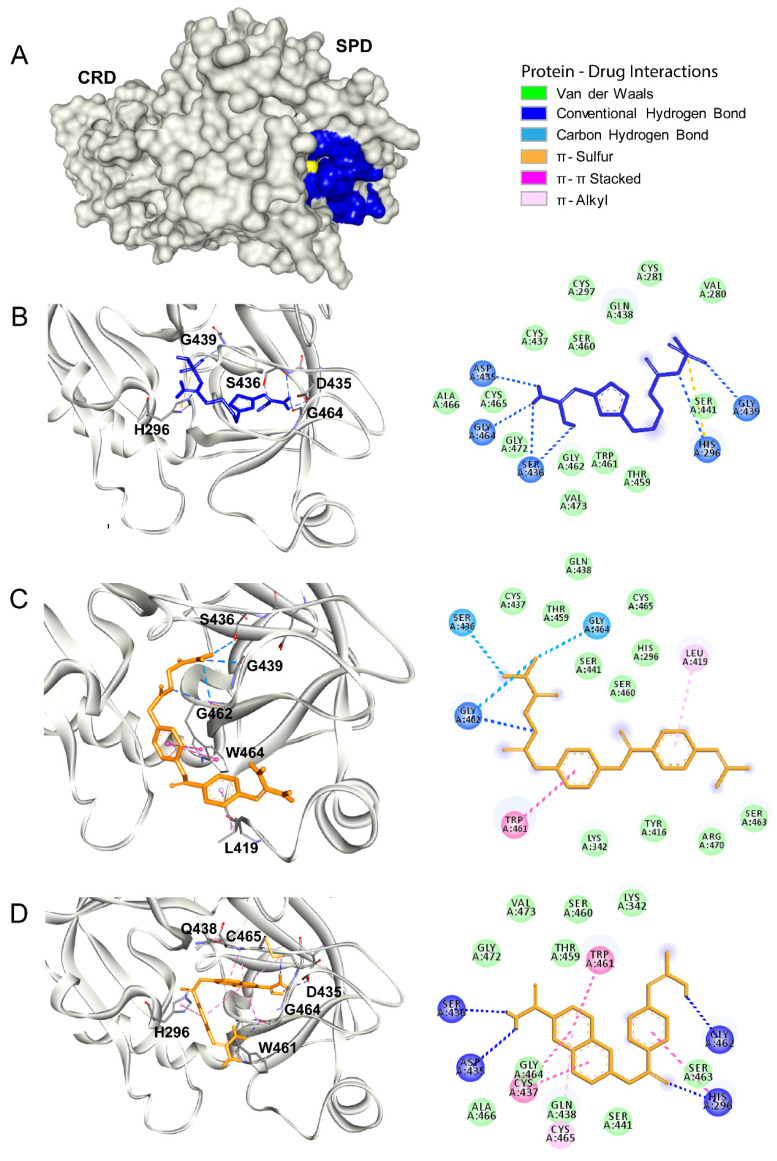
Tmprss2 as a drug target. (**A**) The surface representation of the SARS-CoV2 Tmprss2. The catalytic site is shown in blue, and the catalytic residue Ser441 is shown in yellow. (**B**–**D**) Molecular docking of famotidine, camostat and nafamostat, respectively, into the catalytic site of Tmprss2. The 3D structures of the protein–drug complexes are shown on the left. The protein is represented in ribbon, and drug ligands are shown as sticks. Famotidine is shown in dark blue, while camostat and nafamostat are shown in orange. The main interacting residues are also indicated. The 2D diagrams of the main interaction networks between the drug ligand and the residues within the protease active site are shown on the right. The specific types of interactions are indicated in the legend. The final 3D structures and 2D diagrams of the protein–drug complexes were visualized with the Biovia Discovery Studio Visualizer 17.2.0 software.

The viral replication cycle of SARS-CoV2 starts with an interaction between the viral spike protein and the angiotensin-converting enzyme 2 (ACE2) receptor expressed on the surface of the target host [40]. Then, proteolytic priming occurs in the viral spike protein, allowing the exposure of the fusion motive related to the endosome formation that allows the release of the viral RNA into the host cytosol [7,41]. The human host protease Tmprss2 is involved in this processing of SARS-CoV2 [7]. As reported, protease inhibitors derived from benzoic acid such as nafamostat and camostat showed an antiviral effect against SARS-CoV2 in vitro by blocking Tmprss2 protease activity [7,42]. This protein contains an intracellular domain (residues 1 to 84), transmembrane spanning domain (residues 84–106), low-density lipoprotein receptor domain (LDLRA: residues 133–147), and two extracellular domains. To date, there is no crystal structure solved for Tmprss2. Thus, we developed a homology model by using a crystal structure of a serine protease hepsin (PDB ID: 5CE1) as a template. The homology model of Tmprss2 showed two extracellular domains: the cysteine-rich domain (CRD) (residues 148–242) and the serine protease domain (SPD) (residues 255–489), with the presence of Ser441 as a catalytic residue (Figure 3A). The quality of our homology model was validated using the validation server, and it is depicted with the Ramachandran and the ProSA plots shown in Figure 3B and C. The Ramachandran plot denotes the phi and psi angles of each residue that are related to its structural configuration and indicates if these residues are in an energetically favorable conformation. The assessment of the Ramachandran plot indicates a good overall geometry for the model, with ~98% of the residues in the most favored regions. On the other hand, the ProSA plot contains the *z*-scores of all experimentally determined protein chains in the current PDBs. In this plot, groups of structures from different sources (X-ray, NMR) are distinguished by different colors. Our model has a *z*-score of −8.7 for the overall quality (black dot denoted with yellow circle), which is within the range of scores typically found for native proteins of similar size. Thus, this model is suitable for molecular docking calculations. In our molecular docking analysis, we used antivirals nafamostat and camostat as positive controls (Figure 4C,D, respectively). Both compounds interacted within the active site of Tmprss2 with the following residues: His296, Leu419, Asp435, Ser436, Cys437, Trp461, Gly462, Gly464, and Cys465 via electrostatic interactions and hydrogens bonds. The binding free energy for nafamostat was −7.4 kcal/mol and that for camostat was −6.4 kcal/mol (Table 4). Famotidine interacted with His296, Asp435, Gly439, Ser436, Ser441, and Gly464 within the catalytic site mostly via hydrogen bonds (Figure 4B). The binding free energy for famotidine (−5.8 kcal/mol) (Table 4) was higher than for nafamostat and camostat, indicating its lower affinity to Tmprss2 as compared to two other drugs. The detailed interactions between Tmprss2 and the inhibitors evaluated in this work are shown in the 2D diagrams in Figure 4.

### 3.4. HIV Protease

**Figure 5:**
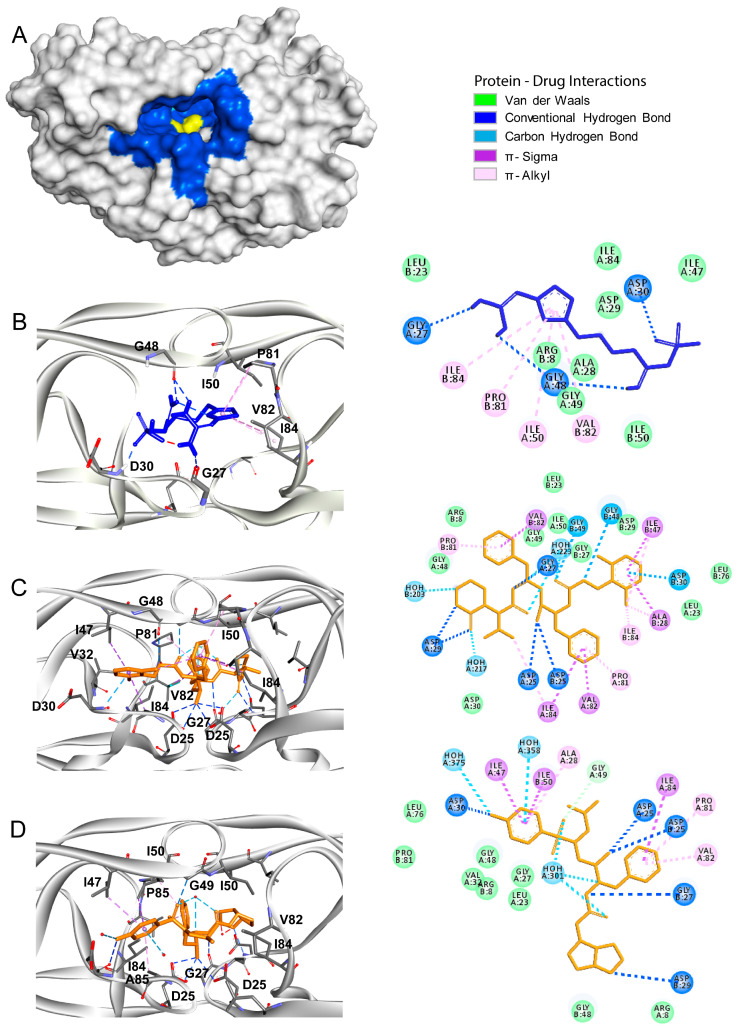
The human immunodeficiency virus (HIV) protease as a target for famotidine. (**A**) The surface representation of the HIV protease crystal structure (PDB ID: 3LZS). The catalytic site is shown in blue and the catalytic residues Asp25, Thr26, and Gly27 are shown in yellow. (**B**) Molecular docking of famotidine into the catalytic site of the HIV protease. The 3D structure of the protein–drug complex is shown on the left. The protein is represented in ribbon, and famotidine is shown as dark blue sticks. The main interacting residues are indicated. The 2D diagrams of the main interaction networks between famotidine and the residues within the protease active site are shown on the right. The specific types of interactions are indicated in the legend. (**C**,**D**) The 3D structures of HIV protease bound to lopinavir (PDB ID: 2Q5K) and to darunavir (PDB ID: 4LL3) obtained from the protein data bank are shown on the left. The 2D diagrams of the main interaction networks between the drug ligand and the residues within the protease active site are shown on the right. The final 3D structures and 2D diagrams of the protein–drug complexes were visualized with the Biovia Discovery Studio Visualizer 17.2.0 software.

Several reports published in the 1990s showed that class A GPCR antagonists, including famotidine, could block HIV replication in vitro [43,44]. However, the exact mechanism related to famotidine inhibition was never determined, mainly due to the approval of more potent HIV inhibitors. Interestingly, HIV has a protease that plays a pivotal role in virus replication by processing the viral polyprotein into mature and functional proteins [45]. The HIV protease is an aspartyl protease and, together with the reverse transcriptase and the integrase, they are the main targets for HIV therapy [46]. There are several drugs approved by the FDA as HIV protease inhibitors. For example, lopinavir and darunavir are commonly used as anti-HIV therapeutics. As described earlier in this report, these two antiviral drugs could bind to the main protease of SARS-CoV2 [42]. We hypothesized that the earlier observed antiviral effect of famotidine against HIV could be related to its interaction with the HIV protease. In order to evaluate this hypothesis, we performed molecular docking of famotidine to the HIV protease. For this analysis, we used the crystal structure of lopinavir-bound HIV protease (PDB ID: 2Q5K) and of darunavir-bound HIV protease (PDB ID: 4LL3) (Figure 5C,D, respectively). Our results indicated that both HIV inhibitors have lower binding free energy (higher affinity) (−10.4 kcal/mol and −10.5 kcal/mol, respectively) than famotidine (−6.4 kcal/mol) (Table 5). The HIV inhibitors produced more electrostatic interactions like π–π stacking and π–alkyl interactions with the active site of HIV protease than famotidine. Nevertheless, famotidine via hydrogen bonds was able to interact with several residues within the active site, including Gly27, which plays a critical role in the structural geometry of the active site (Figure 5B) [47]. The binding poses and the main interactions with the HIV protease for all the examined inhibitors are shown in Figure 5.

### 3.5. Pharmacokinetics versus Pharmacodynamics in Protease Inhibition

All drugs examined in this work could interact with their targets to produce an inhibitory effect. Thus, pharmacodynamically, these drugs should act as inhibitors of the respective proteases in SARS-CoV2. However, often, the results obtained by in silico analyses and in vitro experiments cannot be reproduced in vivo, likely due to drug pharmacokinetic parameters that cannot be fully assessed in the early, experimental stage.

In order to gain a deeper understanding if the pharmacokinetic parameters of the SARS-CoV2 protease inhibitors could be related to positive outcomes in the therapy, we analyzed the ADME parameters of famotidine and compared with several known antiviral drugs such as ribavirin, lopinavir, and nafamostat, which were evaluated against SARS-CoV2. The ADME parameters were determined by using the tools available on the SWISSADME server. The assessment of “drug likeness” in pharmaceutical research is associate with Lipinski’s “rule of five” that established guidelines for the characteristic of permeable compounds through biological membranes. SWISSADME used six physicochemical parameter derivatives from Lipinski’s “rule of five” represented as radar plots of molecule properties. The red area represents “good” property space for oral bioavailability, and the red bold line represents values of calculated properties of the analyzed molecule. When a compound satisfies the “rule of five”, it has molecular properties similar to those of typical bioavailable drugs. However, there are some exceptions from the “rule of five” for certain drug classes (i.e., antibiotics, antifungals, vitamins, and cardiac glycosides).

**Figure 6:**
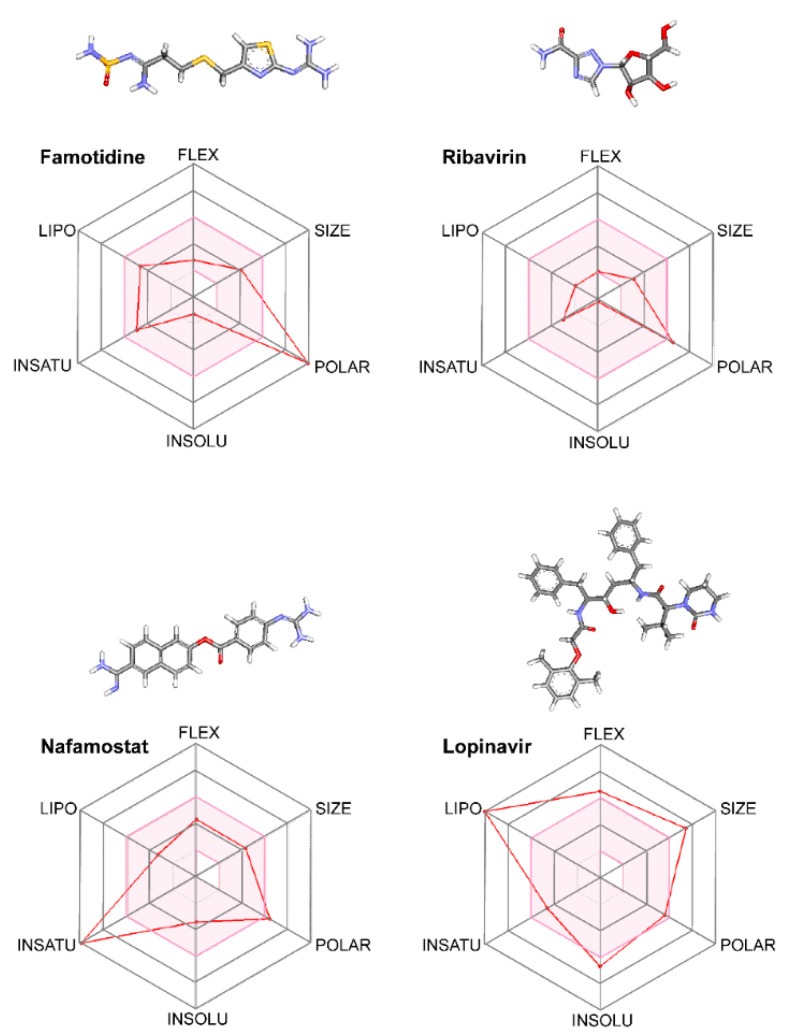
The analysis of pharmacokinetic parameters of protease inhibitors. Chemical structures and administration, distribution, metabolism, and elimination (ADME) parameters for famotidine, ribavirin, lopinavir, and nafamostat, drugs that were evaluated as SARS-CoV2 inhibitors, are shown. The colored zone is a suitable physicochemical space for oral bioavailability obtained using the SWISSADME software. The compounds were scored based on the structure and whether these parameters fit into the values established for each indicator related to oral bioavailability. LIPO (lipophility), SIZE (size), POLAR (polarity), INSOLU (insolubility), INSATU (instauration), and FLEX (flexibility). The 2D structures of each drug are shown with the specific atoms represented as follows: carbon in gray, oxygen in red, nitrogen in blue, and sulfur in yellow.

All the analyzed drugs are FDA-approved and their pharmacokinetics parameters are well known. In Figure 6, we summarize the main ADME parameters in the radar representations for each drug property, including lipophilicity, size, polarity, solubility, saturation, and flexibility. Interestingly, only ribavirin and famotidine exhibited all the properties characteristic for orally available drugs. Of note, famotidine is more polar than ribavirin. All examined compounds except lopinavir have low predicted absorption in the gastrointestinal tract.

The water solubility of a drug is related to its absorption, formulation, and administration pathway. Thus, we evaluated the water solubility of lopinavir, nafamostat, ribavirin, and famotidine by using three predictors. The SWISSADME server uses two topological methods to predict aqueous solubility, the ESOL (Estimated SOLubility) model and the model adapted from Ali et al. [48,49]. The third predictor for solubility was developed by SILICOS-IT. All predicted values are shown as decimal logarithms of the molar solubility in water (log S). The values obtained for each inhibitor are shown in Table 6. Ribavirin and famotidine have higher water solubility with logS (ESL) of −0.21 and −1.25, respectively than lopinavir and nafamostat (logS (ESL) of −6.64 and −3.4, respectively). Lopinavir showed the lowest water solubility among the four analyzed drugs. Interestingly, lopinavir has a very flexible structure and larger size as compared to the other three inhibitors. On the other hand, nafamostat has a higher number of unsaturated bonds than the other analyzed drugs. Both the size and the high content of unsaturated bonds are likely related to the lower solubility of lopinavir and nafamostat.

In the late stage of the disease, the SARS-CoV2 replication occurs mainly in the lungs. Thus, in order to produce a positive effect, an antiviral compound needs to reach the pulmonary tissue. High gastrointestinal absorption of a drug would be advantageous for fast tissue distribution. In fact, FDA-approved drugs currently used for treatment of SARS-CoV2 such as chloroquine, camostat, and lopinavir have high gastrointestinal absorption. However, in the case of lopinavir, reaching the pulmonary tissue could be delayed due to its high volume of distribution, which is associated with its tight binding to plasma proteins [50,51,52,53]. On the other hand, famotidine has low gastrointestinal absorption; however, due to its high polarity, it could be given intravenously. The intravenous route of drug administration increases the probability of reaching the tissue target. Thus, if administered intravenously, famotidine has a better chance to be a successful anti-SARS-CoV2 drug in the late stage of infection than lopinavir. Thus, in order to develop efficient anti-SARS-CoV2 therapy, the pharmacokinetic parameters associated with drug absorption, metabolism, and distribution need to be carefully taken into account.

## 4. Discussion

Initially, SARS-CoV2 antiviral treatment strategies were adopted based on the prior clinical approaches used for combating the viral respiratory infections, mainly the SARS-CoV infection. These strategies included treatments with interferon-alpha, lopinavir/ritonavir combination, ribavirin, and chloroquine [54,55,56]. However, the outcomes of these treatments were not as good as expected, mainly due to low viral clearance and side effects. Thus, multiple research groups currently focus on basic research approaches using in silico and in vitro screenings to quickly find new therapeutic alternatives [57,58]. In a pandemic situation, time is crucial. Thus, adopting already existing drugs for identifying new functions of these drugs, known as drug repurposing, could decrease the time and cost of developing new therapy [15]. The screening of large libraries of compounds already approved by the FDA against the main targets in SARS-CoV2 can assure an accelerated start to clinical trials [59].

The SARS-CoV2 replication cycle offers several steps that could be used as molecular targets for antiviral drugs. The most evaluated are viral entry, the main protease, the papain-like protease, and the RNA-depended RNA polymerase [60,61,62]. The computational and in vitro analyses of these targets and specific antiviral drugs are ongoing and some were already reported [33]. Based on the results of these studies, some clinical trials of antiviral drugs against SARS-CoV2 are currently in progress. In this study, by using computational analyses, we examined the therapeutic potential of famotidine as an anti-SARS-CoV2 drug. Structurally, famotidine is a member of 1,3-thiazoles, a sulfonamide, and a member of guanidines. This histamine type 2 receptor antagonist was never tested as an antiviral agent against Coronavirus. However, as reported in the 1990s, famotidine could inhibit HIV replication in vitro, but its mechanism of action was never clarified [43,44]. Recently, in silico analysis of the SARS-CoV2 targets and their potential new inhibitors suggested that famotidine could possibly target the SARS-CoV2 main protease [33]. Thus, in this study, we analyzed if famotidine could also inhibit other proteases involved in the viral replication and, thus, similarly to ribavirin, act as a broad-antiviral agent. Ribavirin, a purine nucleoside analogue demonstrated efficacy against variety of DNA and RNA viral infections [33,63]. The overwhelming success of ribavirin is largely related to its excellent performance in synergy with standard or pegylated interferon-alpha in chronic hepatitis C virus (HCV) infection [64,65]. Our in silico analysis revealed that famotidine can interact with the SARS-CoV2 main protease with a binding free energy of −6.4. Moreover, it could also interact with two other proteases involved in SARS-CoV2 replication, the viral PLpro and human host Tmprss2. However, the binding free energies for these proteases (−5.0 and −5.8, respectively) were higher (lower affinities) than for the main protease. Nevertheless, these results indicate that famotidine could bind non-specifically to all proteases involved in virus replication with rather low binding affinity. Thus, it seems that, similarly to ribavirin, the best therapeutic effect of famotidine could be achieved in combination therapy with other antiviral drugs [64,65]. The binding affinity of famotidine to the viral and host proteases could be related to its chemical structure that lacks multiple aromatic rings as compared with other inhibitors, thus showing higher binding free energy (Table 1, Table 2, Table 3 and Table 4). These aromatic rings produce electrostatic interactions such as π–π interactions and π–alkyl interactions with the residues in the catalytic site of the enzyme that stabilize the compound and decrease the binding free energy. The molecular docking score functions are used to assign a binding free energy value that could be in relation to the binding efficacy of the molecule to the active site. In this work, we refer to this efficacy as an affinity for the binding site. However, further in vitro assays are necessary to validate this data and to establish a correlation between the affinity values and experimental drug binding constants for each enzyme. Nevertheless, the high binding free energy of famotidine reflects its low target specificity. Thus, to enhance the potential positive outcome of famotidine therapy, it is highly likely that it has to be administered together with other antiviral drugs.

The combination of famotidine and hydroxychloroquine could be a valid approach to treat SARS-CoV2 infection. Hydroxychloroquine elevates the pH of acidic intracellular organelles, such as endosomes and lysosomes, essential for membrane fusion. Thus, this drug could inhibit endosome-mediated viral entry [66,67]. In addition, pH elevation impairs enzymes involved in protein posttranslational modifications. Indeed, hydroxychloroquine could inhibit SARS-CoV cellular entry through changing the glycosylation pattern of ACE2 receptor and spike protein [68]. Thus, hydroxychloroquine is a non-specific antiviral drug that could improve the effectiveness of other antiviral drugs when administered simultaneously. Multiple studies evaluating a combination of hydroxychloroquine with other antiviral drugs are ongoing (NCT04370782, NCT04374019, NCT04373044). For example, a small open-labeled, non-randomized clinical trial from France, evaluating hydroxychloroquine, demonstrated its positive effect in combination with azithromycin [69]. However, larger randomized controlled clinical trials are needed to attain deeper knowledge about the positive effect of these drugs combination. Another clinical trial evaluating hydroxychloroquine in combination with camostat recently began in Germany (NCT04338906). A combination of these two drugs should produce a synergistic effect by inhibiting two main mechanisms associated with the early stage of viral entry. A combination therapy of hydroxychloroquine with famotidine could possibly achieve synergy by blocking both the early infection stage with hydroxychloroquine and the late stage with famotidine.

The analysis of pharmacokinetic parameters of famotidine revealed that the anti-SARS-CoV2 effect of this drug could likely be achieved only if famotidine is administered intravenously. Famotidine exhibits low gastrointestinal absorption and it has a low volume of distribution; thus, if given orally, it would most likely not be able to reach the target respiratory pathway to produce an antiviral effect. Therefore, drugs with low gastrointestinal absorption and low volume of distribution such as famotidine and nafamostat should be given via intravenous injection in the clinical setup. On the other hand, compounds such as hydroxychloroquine or ribavirin that have high gastrointestinal absorption and high volume of distribution [53] should achieve therapeutic effect upon oral administration. Unexpectedly, anti-HIV drug lopinavir, despite its high gastrointestinal absorption, proved to be ineffective against SARS-CoV2 infection. The poor outcome of lopinavir treatment could be related to other pharmacokinetic parameters of this drug such as high metabolism and low bioavailability [17,53]. Although the volume of distribution of lopinavir upon its oral administration is reasonably small (~16.9 L) [53], this drug tightly binds to plasma proteins (over 90%) [53]. Thus, its plasma concentration of free drug is likely too low to reach the pulmonary tissue to target SARS-CoV2. Thus, in order to develop successful effective antiviral therapy targeting SARS-CoV2, the pharmacokinetic parameters should be carefully taken into consideration.

## 5. Conclusions

Altogether, in this study, we showed that famotidine could be used as an antiviral agent against SARS-CoV2, targeting proteases involved in the virus replication, mostly the main protease, as well as the viral PLpro and human host Tmprss2. Based on our analysis, it is unlikely that famotidine would produce a strong antiviral effect. It is rather expected that famotidine should be administered together with other antiviral drugs in order to produce synergistic effect to combat SARS-CoV2 infection. Although our results obtained using in silico analysis need further experimental validation, the potential of famotidine as an inhibitor of proteases implicated in viral replication is already supported by the published data obtained from retrospective studies, which showed the beneficial effect of famotidine against SARS-CoV2. In addition, one clinical trial aiming to evaluate the safety and effectiveness of famotidine against SARS-CoV2 is already ongoing. However, taking into account the pharmacokinetic parameters of famotidine, we predict that it could only be effective as an anti-SARS-CoV2 drug when given intravenously in the clinical health facility by healthcare providers.

## Figures and Tables

**Table 1:** Chemical structure of the compounds evaluated as protease inhibitors.

Compound	Chemical Structure
Name: Famotidine Chemical formula: C_8_H_15_N_7_O_2_S_3_ Molecular Weight: 337.44 g/mol	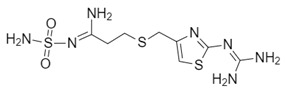
Name: Lopinavir Chemical formula: C_37_H_48_N_4_O_5_ Molecular Weight: 628.80 g/mol	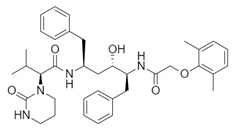
Name: Nafamostat Chemical formula: C_19_H_17_N_5_O_2_ Molecular Weight: 347.37 g/mol	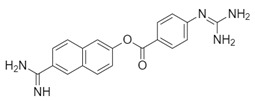
Name: Ribavirin Chemical formula: C_8_H_12_N_4_O_5_ Molecular Weight: 244.20 g/mol	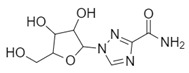
Name: SARS-CoV(1) Chemical formula: C_25_H_27_FN_2_O Molecular Weight: 390.49 g/mol	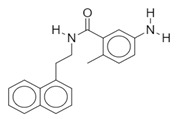
Name: SARS-CoV(2) Chemical formula: C_20_H_20_N_2_O Molecular Weight: 304.38 g/mol	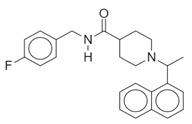
Name: Darunavir Chemical formula: C_27_H_37_N_3_O_7_S Molecular Weight: 547.7 g/mol	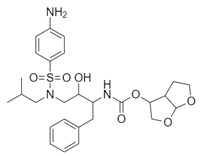
Name: Camostat Chemical formula: C_20_H_22_N_4_O_5_ Molecular Weight: 398.4 g/mol	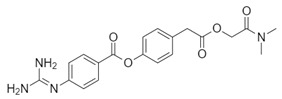

**Table 2:** Binding free energies calculated for famotidine and other protease inhibitors upon docking to the SARS-CoV2 main protease.

Compound	Pubmed ID	Binding Free Energy (kcal/mol)
Famotidine	24883443	−6.4
Lopinavir	92727	−9.0
Darunavir	213039	−8.3

**Table 3:** Binding free energies calculated for famotidine and other protease inhibitors upon docking to the SARS-CoV2 papain-like protease.

Compound	Pubmed ID	Binding Free Energy (kcal/mol)
Famotidine	24883443	−5.0
Ribavirin	37542	−6.1
SARS-CoV(1)	44828571	−6.5
SARS-CoV(2)	73659185	−6.6

**Table 4:** Binding free energies calculated for famotidine and other protease inhibitors upon docking to human host protease Tmprss2.

Compound	Pubmed ID	Binding Free Energy (kcal/mol)
Famotidine	24883443	−5.8
Nafamostat	4413	−7.6
Camostat	2536	−6.3

**Table 5:** Binding free energies calculated for famotidine and other protease inhibitors upon docking to HIV protease.

Compound	Pubmed ID	Binding Free Energy (kcal/mol)
Famotidine	24883443	−6.4
Lopinavir	92727	−10.4
Darunavir	213039	−10.5

**Table 6:** Water solubility of drugs used as inhibitors of SARS-CoV2 proteases.

Solubility Predictor	Famotidine	Ribavirin	Lopinvavir	Nafamostat
Log S (ESOL)	−1.25	−0.21	−6.64	−3.40
Solubility	5.60 × 10^−2^ M	6.19 × 10^−1^ M	2.31 × 10^−7^ M	4.00 × 10^−4^ M
Class	Very soluble	Very soluble	Poorly soluble	Soluble
Log S (Ali)	−3.88	−0.65	−8.21	−4.61
Solubility	1.32 × 10^−4^ M	2.24 × 10^−1^ M	6.10 × 10^−9^ M	2.46 × 10^−5^ M
Class	Soluble	Very soluble	Poorly soluble	Moderately soluble
Log S (SILICOS-IT)	−1.26	1.76	−10.05	−5.35
Solubility	5.43 × 10^−2^ M	5.73 × 10^1^ M	8.85 × 10^−11^ M	4.46 × 10^−6^ M
Class	Soluble	Soluble	Insoluble	Moderately soluble

ESOL, Topological method implemented from Delaney [48]; Ali, Topological method implemented from Ali et al. [49]; SILICOS-IT, Fragmental method calculated FILTER-IT program.

## Abbreviations

3CLpro	SARS-CoV2 main protease
ACE2	angiotensin-converting enzyme 2
ADME	administration, distribution, metabolism, and elimination
COVID-19	Coronavirus infectious disease
CRD	cysteine-rich domain
FDA	Food and Drug Administration
FLEX	flexibility
GPCR	G protein-coupled receptor
HIV	human immunodeficiency virus
INSOLU	insolubility
INSATU	instauration
LDLRA	low-density lipoprotein receptor domain
LIPO	lipophilicity
NMR	nuclear magnetic resonance
PDB	Protein Data Bank
PLpro	SARS-CoV2 papain-like protease
POLAR	polarity
SARS-CoV2	Severe Acute Respiratory Syndrome Coronavirus type 2
SARS-CoV	Severe Acute Respiratory Syndrome Coronavirus type 1
SPD	serine protease domain
Tmprss2	transmembrane serine protease type II
